# Extensive Colonic Pneumatosis, Pneumoperitoneum, Pneumomediastinum and Subcutaneous Emphysema – a Rare Pattern of Complications in Acute Lymphoblastic Leukemia

**DOI:** 10.5334/jbsr.2785

**Published:** 2022-04-25

**Authors:** Ana T. Vilares, Bárbara Viamonte, António J. Madureira

**Affiliations:** 1 Centro Hospitalar Universitário de São João, PT

**Keywords:** Colonic Pneumatosis, Pneumoperitoneum, Pneumomediastinum, Acute Lymphoblastic Leukemia, Treatment Complications

## Abstract

**Teaching Point:** Intestinal pneumatosis associated with pneumoperitoneum, pneumomediastinum, and subcutaneous emphysema is an extremely rare complication of leukemia; even though its imaging appearance can be alarming, a benign treatment-associated etiology should always be considered in this subset of patients so that unnecessary interventions can be avoided.

## Case History

**Figure 1:**
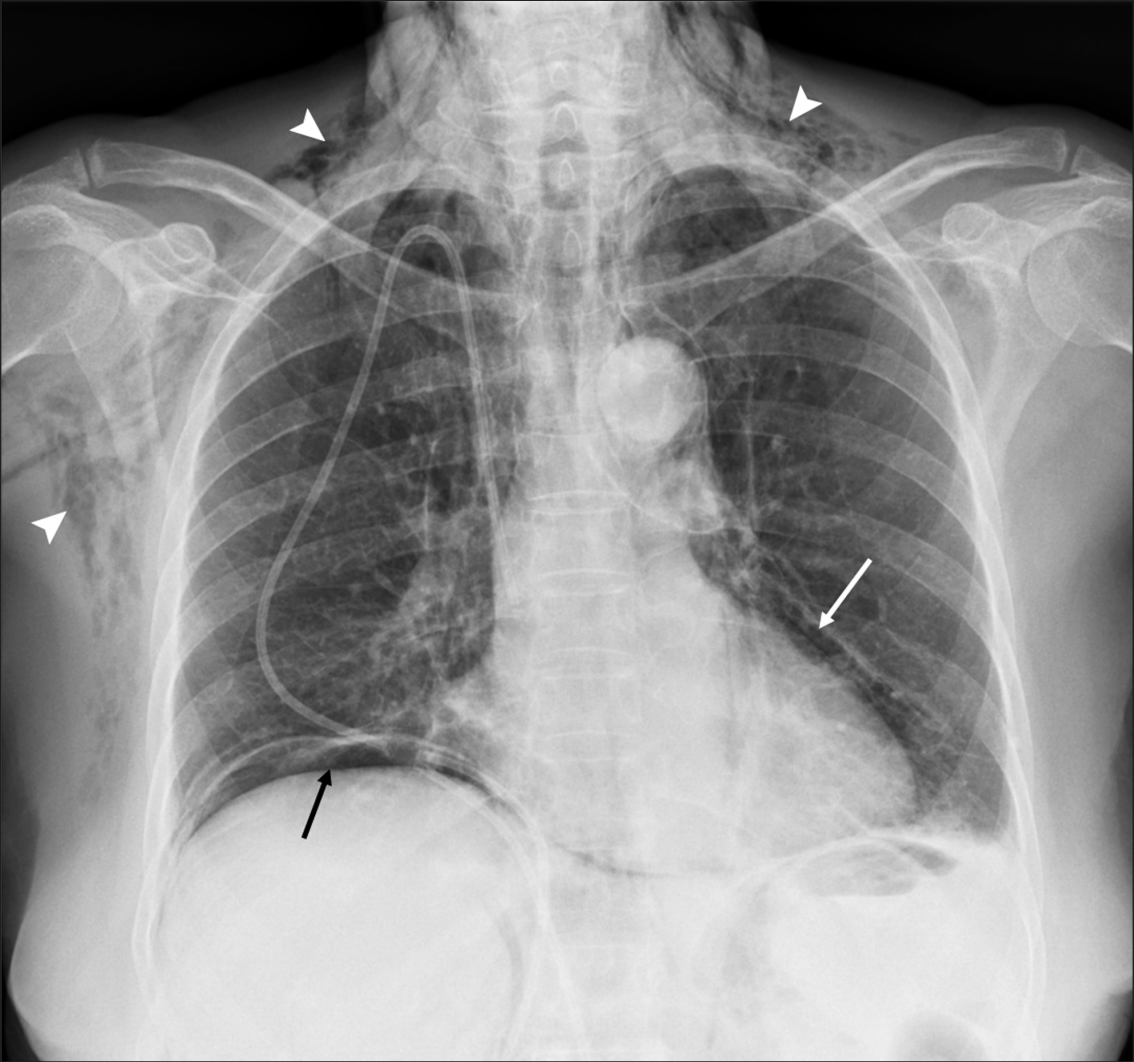


A previously healthy, 69-year-old female was admitted to the hemato-oncology department with a new-onset diagnosis of BCR-ABL+ acute lymphoblastic leukemia B. Treatment with standard induction chemotheraphy and Imatinib was initiated. Twenty-five days later the patient developed dyspnea and nausea complaints. Vital parameters were normal. Pulmonary auscultation revealed diffuse crepitations. The abdomen was distended and diffusely tender, without signs of peritoneal irritation. Laboratory studies showed mild PCR elevation. A chest radiograph (CXR) was obtained (***Figure 1***), revealing pneumomediastinum (white arrow), pneumoperitoneum (black arrow), and subcutaneous emphysema (arrowheads).

**Figure 2:**
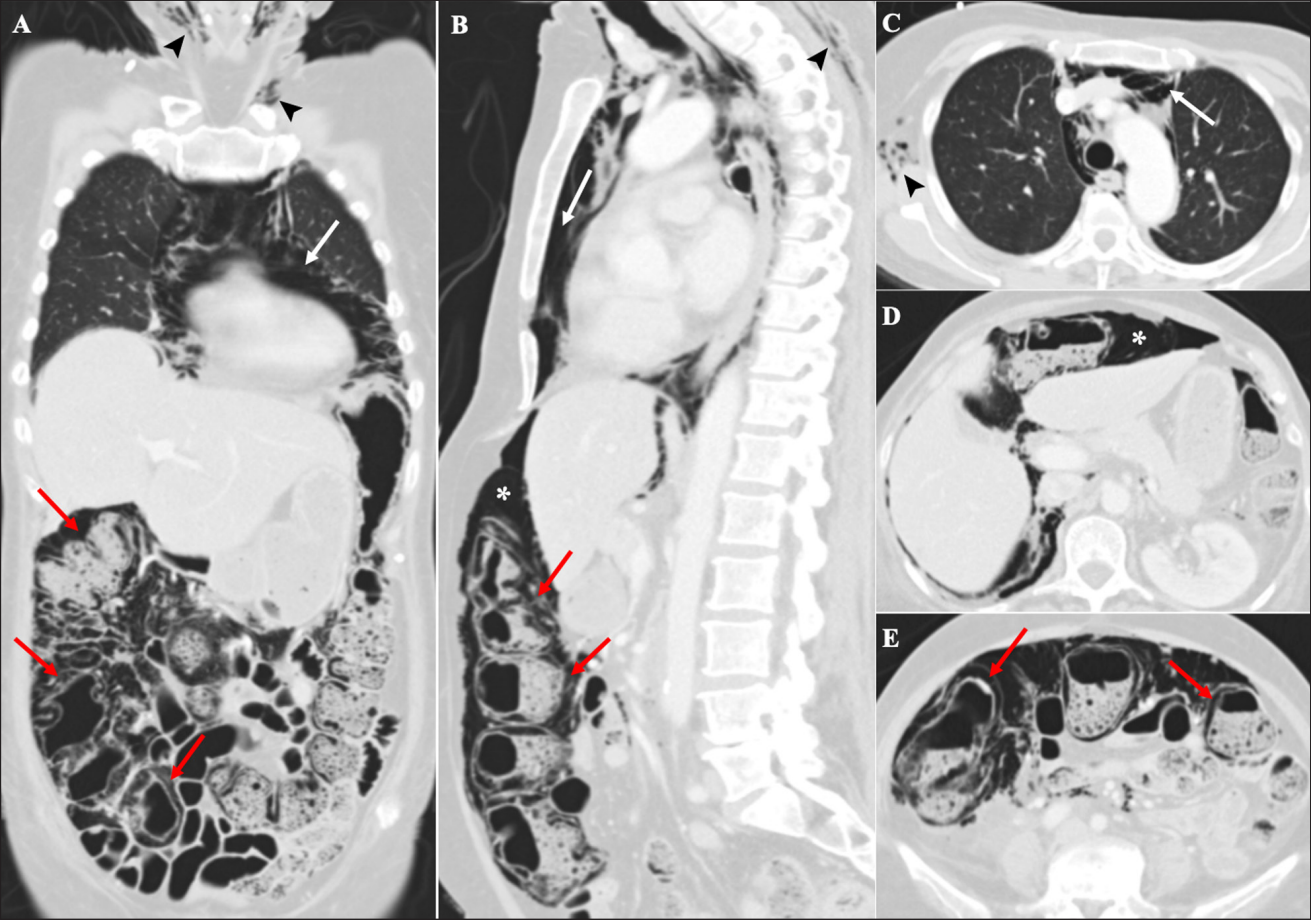


A Computed Tomography (CT) (***Figure 2***) was then requested, revealing extensive pneumatosis of the right and transverse colon (red arrows), pneumoperitoneum (asterisks), pneumomediastinum (white arrows), and subcutaneous emphysema (arrowheads). No evidence of gastrointestinal perforation was found on CT. The mesenteric vessels were patent and indirect signs of bowel ischemia, such as ascites or portal pneumatosis, were also absent.

**Figure 3:**
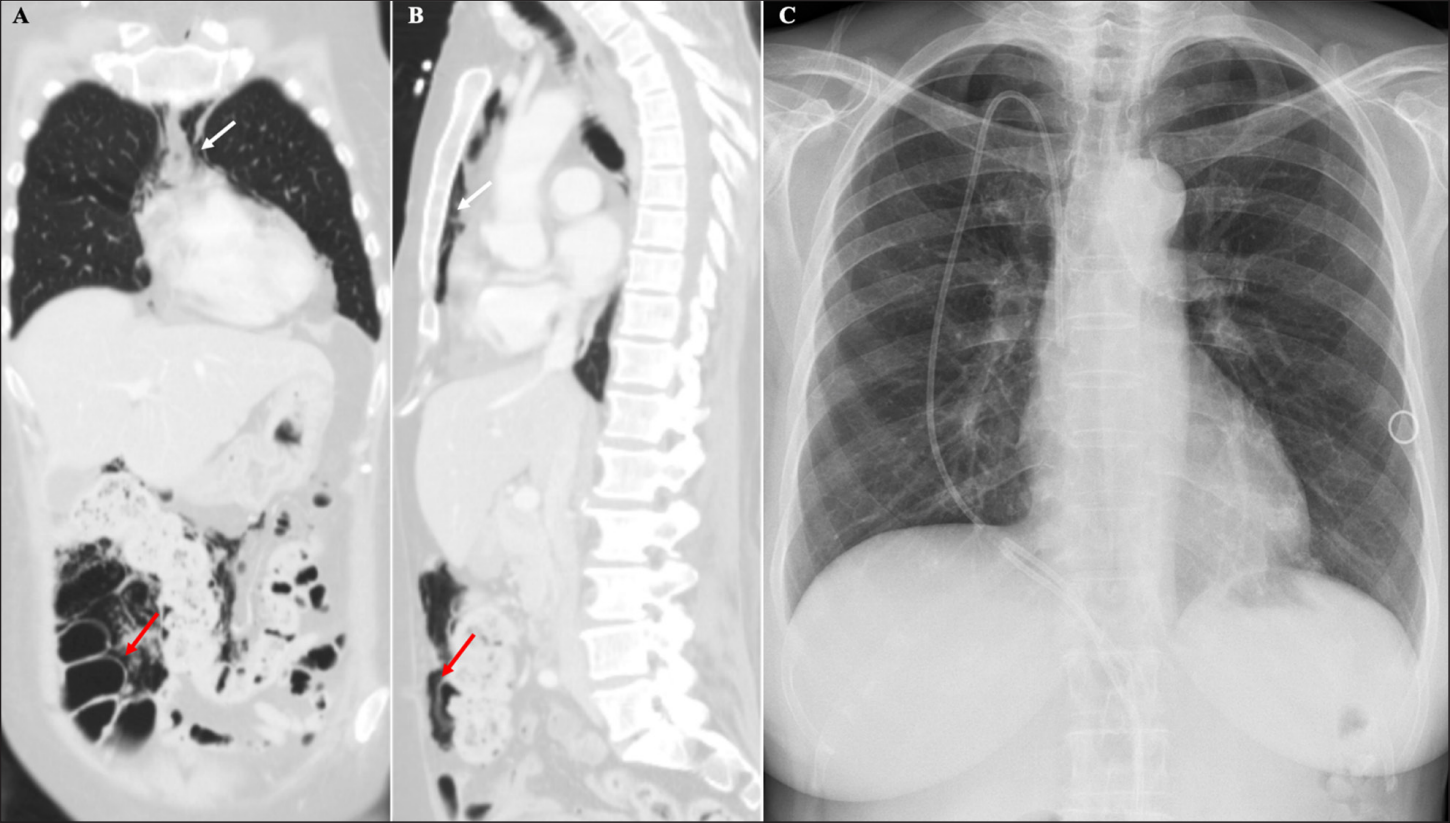


Surgical exploration to definitely exclude GI perforation was initially considered. However, since the surgical risk was significative and the patient was stable, both the medical and surgical teams agreed to proceed with a conservative approach. The patient was admitted to an intermediate care unit and remained under close clinical surveillance; supplemental oxygen, bowel rest, and broad-spectrum antibiotic therapy were initatiated. Imatinib and corticosteroids were discontinued. The patient remained stable, and the dyspnea and abdominal pain complaints progressively resolved. CT was repeated three days later, revealing marked reduction of the colonic pneumatosis (red arrows) and pneumomediastinum (white arrows) (***Figure 3A, B***). Resolution of the pneumoperitoneum and pneumomediastinum was documented on CXR performed two days later (***Figure 3C***). The patient was then readmitted to the hematology department to proceed with therapy.

## Comment

Intestinal pneumatosis (IP) associated with pneumoperitoneum is generally regarded as an alarming radiological feature, suggestive of underlying bowel disease, such as GI perforation or mesenteric ischemia. However, it can also be observed in severely immunocompromised oncologic patients, in the absence of bowel pathology [1]. In this subset of patients, the etiology of IP is multifactorial, resulting from altered intestinal permeability, mucosal defects, and immunosuppression from corticosteroids and chemotherapy [1]. Recent evidence suggests that tyrosine kinase inhibitors, including Imatinib, can also induce IP.

The development of pneumoperitoneum is thought to result from the rupture of bowel wall pneumatoceles, while pneumomediastinum and subcutaneous emphysema, albeit rarer, can occur if there is air leakage from the peritoneal cavity into the mediastinum and cervical tissues.

When worrisome clinical and radiological findings are absent, these patients can be treated conservatively with a high-success rate.
